# Molecular and immunological mechanisms of clonal evolution in multiple myeloma

**DOI:** 10.3389/fimmu.2023.1243997

**Published:** 2023-09-06

**Authors:** Stefan Forster, Ramin Radpour, Adrian F. Ochsenbein

**Affiliations:** ^1^Tumor Immunology, Department for BioMedical Research (DBMR), University of Bern, Bern, Switzerland; ^2^Department of Medical Oncology, Inselspital, Bern University Hospital, University of Bern, Bern, Switzerland

**Keywords:** multiple myeloma, malignant plasma cell, clonal evolution, tumor microenvironment, TME, immunotherapy, drug resistance

## Abstract

Multiple myeloma (MM) is a hematologic malignancy characterized by the proliferation of clonal plasma cells in the bone marrow (BM). It is known that early genetic mutations in post-germinal center B/plasma cells are the cause of myelomagenesis. The acquisition of additional chromosomal abnormalities and distinct mutations further promote the outgrowth of malignant plasma cell populations that are resistant to conventional treatments, finally resulting in relapsed and therapy-refractory terminal stages of MM. In addition, myeloma cells are supported by autocrine signaling pathways and the tumor microenvironment (TME), which consists of diverse cell types such as stromal cells, immune cells, and components of the extracellular matrix. The TME provides essential signals and stimuli that induce proliferation and/or prevent apoptosis. In particular, the molecular pathways by which MM cells interact with the TME are crucial for the development of MM. To generate successful therapies and prevent MM recurrence, a thorough understanding of the molecular mechanisms that drive MM progression and therapy resistance is essential. In this review, we summarize key mechanisms that promote myelomagenesis and drive the clonal expansion in the course of MM progression such as autocrine signaling cascades, as well as direct and indirect interactions between the TME and malignant plasma cells. In addition, we highlight drug-resistance mechanisms and emerging therapies that are currently tested in clinical trials to overcome therapy-refractory MM stages.

## Introduction

Multiple Myeloma (MM) belongs to the heterogeneous group of plasma cell disorders comprising monoclonal gammopathy of undetermined significance (MGUS), smoldering myeloma (SMM), plasma cell myeloma, plasmacytoma, monoclonal immunoglobulin deposition disease and plasma cell neoplasms associated with paraneoplastic syndromes (1). Plasma cell myeloma/MM is the most frequent plasma cell disorder and is characterized by the uncontrolled expansion of malignant plasma cells in the bone marrow (BM) causing distinct clinical symptoms such as bone pain, spontaneous fractures, and renal impairment (1, 2). To date, MM is still considered incurable. However, due to the introduction of novel immunotherapies that directly target plasma cell surface markers such as CD38, the overall and progression-free survival of myeloma patients has substantially improved within the last years (3–5). For the diagnosis of MM, the international myeloma working group (IMWG) requires 10% BM plasma cells or a biopsy-proven bone or extramedullary plasmacytoma with at least one or more CRAB criteria (calcium elevation, renal insufficiency, anemia, bone lesions) and/or myeloma-defining molecular aberrations (6). MGUS and SMM are considered as the precursor lesions of MM (2, 6, 7). Patients with MGUS and SMM have increased BM plasma cell counts and/or monoclonal proteins, but lack MM-defining symptoms (6, 7). Early genetic alterations in the malignant transformation process include hyperdiploidy or translocations involving the immunoglobulin heavy chain (IGH) locus on chromosome 14 that lead to the overexpression or overactivation of defined oncogenes stimulating plasma cell proliferation and/or preventing apoptosis (8–11).

As a consequence of early cytogenetic alterations that occur in postgerminal center B/plasma cells, a small group of abnormal cells, so called founder clones, expand and initiate the process of myelomagenesis. Subsequently, the clonal evolution of malignant plasma cells emerges through a series of additional copy number modifications, epigenetic changes, and the acquisition of additional secondary mutations that enhance the intratumoral heterogeneity finally resulting in the co-existence of multiple clonal subpopulations with various selection and/or fitness advantages (2, 12). Indeed, the spatio-temporal clonal landscape drastically changes in the course of MM progression thus favoring disease-promoting clones during repetitive cycles of myeloma cell engraftment, dissemination and re-engraftment at another BM site, as previously shown for MM xenografts grown in severe combined immunodeficiency (SCID) mice (12). Moreover, so called focal lesions - clonally heterogeneous and spatially distributed tumor clusters - are seen as mutational “hot spots” in MM, consistent with the regional outgrowth of advanced tumor clones (13, 14). In relapsed and therapy-refractory MM end-stages, single myeloma clones might even lose their BM dependency, survive and expand in the circulation, or spread to distant body regions, resulting in plasma cell leukemia or extramedullary myeloma (15, 16). Both, plasma cell leukemia and extramedullary myeloma are defined by rapid disease onset, poor therapy response, and an overall poor prognosis (15).

Multiple molecular mechanisms might influence the outgrowth of MM clonal subsets. Based on specific cytogenetic alterations that occur in clonal plasma cells during MM progression, myeloma cells may develop resistance to standard MM treatments such as proteasome inhibitors and immunomodulatory agents driving the selection of drug-resistant populations that outcompete drug-sensitive populations. Furthermore, the activation of autocrine signaling loops might inhibit tumor cell death or enhance cell proliferation of clonal subsets that co-express the corresponding ligand-receptor pairs. Finally, the cross-talk between tumor cells and the tumor microenvironment (TME) promotes the dissemination of distinct clonal populations that are seen as the main drivers of MM progression (12). In summary, this review outlines the central pathways and mechanisms that drive myelomagenesis and contribute to the clonal evolution and expansion of malignant plasma cells finally leading to MM progression and therapy-refractory end-stages.

## MM stem cells/stem cell-like cells

Cancer stem cells (CSCs) are a small subpopulation of cancer cells harboring an unlimited capacity of self-renewal. Low proliferation or quiescence make CSCs resistant to radiation and chemotherapies that predominantly target rapidly reproducing cancer cells. As a result, CSCs are often considered crucial for disease recurrence (17–20). CSCs have been identified in a variety of cancer entities including colorectal cancer, lung cancer, pancreatic cancer and acute myeloid leukemia (21–26). However, the presence and (immuno-) phenotypic characterization of MM CSCs or cancer-stem cell like cells have been controversially discussed in the past (27). So far, no particular cellular or molecular markers have been identified to accurately distinguish myeloma CSCs from the remaining tumor mass. Plasma cells, both normal and malignant, are terminally differentiated cells. Thus, myeloma stem cells are thought to be derived from abnormal postgerminal center B cells (27, 28). Rasmussen et al. discovered clonotypic memory B cells in most MM patients (29). In immunodeficient mice, injection of blood-derived CD19^+^ CD27^+^ B cells from myeloma patients successfully initiated the disease, whereas CD138^+^ plasma cells failed to engraft *in vivo* (30). Myeloma-derived CD19^+^ CD138^-^ leukemic cells engrafted in NOD/SCID mice, indicating a role of clonotypic late-stage B-cells in disease initiation (31). In line, RPMI8226 and NCI H929 cell line derived CD138^-^ cell fractions had increased ALDH1 enzyme activity and superior clonogenic potential both *in vitro* and *in vivo* (32). In colony formation assays – an approach to investigate clonogenicity, stemness and self-renewal – MM cell line and patient derived CD138^-^ fractions showed increased colony formation upon serial replatings compared to CD138^+^ fractions (33). In contrast, other studies postulated that myeloma stem cells are characterized by a CD38^+^ CD138^+^ CD19^-^ CD45^-^ immunophenotype suggesting that stem cell related markers might undergo dynamic changes or differ between MM patients and the experimental model systems (34, 35). Indeed, environmental factors influence the expression of distinct plasma cell surface molecules. Xenografts created by the injection of CD19^+^ CD138^-^ myeloma cell fractions showed partial CD138 re-expression in the primary engrafted tumor, whereas CD138 expression was almost non-existent in circulating B cells, suggesting that CD138 expression is dependent on environmental cues (30, 31). Nutrient deprivation changes CD138 surface expression, and CD138^+^ Vk*MYC murine myeloma cells showed better engraftment and tumor development, whereas CD138^-^ cells were characterized by increased motility, intravasation, and dissemination (36). In addition, low oxygen levels decrease CD138 expression and induce a mature B cell-like transcriptional signature with upregulation of *PAX5* and *BCL6* in myeloma cells, as previously described by Kawano et al. (37). Hypoxia resulted in the upregulation of the stem cell-related transcription factors *OCT4* and *SOX2* in the MM cell line RPMI8226 (37).

A small side population (SP) of clonogenic myeloma cells has been revealed by reduced Hoechst 33342 staining (30, 38, 39). Multiple independent studies demonstrated that SP cells harbor stem cell like features with decreased drug sensitivity and over-activation of stem cell related pathways including Notch-, Hedgehog-, PI3K/Akt or Wnt-signaling that are also found enriched in most MM patients (40–50). In line with previous findings, Wang et al. reported that SP myeloma cells are more resistant to bortezomib or melphalan chemotherapy and that SP abundance and clonogenicity are regulated by the activated-leukocyte-cell-adhesion-molecule (ALCAM)/EGFR-EGF signaling axis (49, 51). Interestingly, combination therapy with melphalan and an EGFR inhibitor resulted in a reduction of the SP and a significantly lower disease burden in 5TGM1 myeloma bearing mice compared to monotherapies (49). Furthermore, the expression of specific molecules such as CD24, CD34 and ATP binding cassette subfamily G member 2 (ABCG2) have been linked to a MM stem cell-like phenotype (52–54). CD24 expression is increased in MM SP and the stem cell related genes *KLF4*, *OCT4*, *NANOG* and *SOX2* were enriched in isolated CD24^+^ cells. *In vivo* limiting dilution assays revealed that CD24^+^ myeloma cells showed a significantly higher tumor initiating capacity in NOD-Rag1null mice compared to CD24^-^ fractions (55).

While the concept of MM founder clones – defined as early mutation harboring clones that initiate myelomagenesis - is widely accepted and supported by numerous research studies - no unified characterization and terminology of MM CSCs – that maintain and propagate the disease - have been reached (27, 56, 57). In this regard, high cancer cell plasticity with dynamic expression profiles of stem-cell related markers – in parts driven by environmental conditions - might be a fundamental challenge in defining and eventually targeting MM CSCs.

## Genetic and epigenetic instability drives the clonal evolution starting from early founder clones

The clonal evolution of MM occurs through a series of genetic alterations, including chromosomal abnormalities, somatic mutations, and epigenetic modifications. Early genetic events are divided in two groups. (1) Translocations involving the IGH locus on chromosome 14 such as t(11;14), t(4;14), t(14;16) leading to the overexpression of the oncogenic drivers *CCND1*, *MMSET*/*FGFR3* or *cMAF*, respectively. Overexpression of the *MMSET*/*FGFR3* fusion gene is caused by the translocation t(4;14), one of the most important chromosomal abnormalities in MM (58–64). (2) Hyperdiploidy of the odd-numbered chromosomes 3, 5, 7, 9, 11, 15 or 19 might also trigger myelomagenesis (58, 65). Moreover, single-cell RNA-sequencing and single-molecule long-read sequencing revealed early *IFITM2* and *ANK1* alterations in clonal plasma cells (66). Multiple evolutionary branches emerge from a single abnormal postgerminal center B-/plasma cell during MM development, driven in part by TME-related interactions (67). In this regard, the clonal evolution of abnormal plasma cells is thought being initiated by genetic mutations occurring in postgerminal center B cells in the course of somatic hypermutation and isotype class switching. After homing to the BM, these mutation-harboring B-cells become so called founder clones that start the clonal evolution process (56, 57). Interestingly, the number of founder clones and the patterns of clonal evolution differs in-between patients. Whole exome sequencing of paired biopsies at diagnosis and progression revealed that in most cases two founder clones are present at disease onset (range one to three founder clones) (67, 68). After disease initiation, the clonal evolution most often follows a branching pattern, where multiple heterogeneous clones develop independently and distinct mutations are lost or gained in the course of MM progression. Less frequently, the clonal structure persists between early and late disease stages, referred as a stable evolutionary pattern (69, 70). Notably, clonal heterogeneity can be found throughout all steps of myeloma progression starting from MGUS (71, 72). CyTOF analyses of distinct markers involved in B cell regulation (e.g. MMSET or sXBP1), stemness (e.g. SOX2) and abnormal plasma cell differentiation (e.g. CD56) revealed different clusters among the B cell/plasma cell compartment and heterogeneous expression profiles within these defined clusters, in-between patients and among MGUS, SMM, MM or relapsed MM stages (72). Longitudinal whole-exome sequencing analyses of plasma cells from paired MGUS/SMM and MM patients revealed intraclonal heterogeneity at the early MGUS/SMM stages characterized by the detection of multiple co-existing clonal subsets each harboring a distinct set of driver gene mutations (71). Further, Dutta et al. described a subclonal stability between MGUS/SMM and MM stages – meaning that the subclonal architecture and heterogeneity found at advanced disease stages is often already present at MGUS/SMM (71). After disease onset with MM manifestation, the further clonal evolution of malignant plasma cell subsets is driven by late stage (or secondary) somatic mutations involving *KRAS*, *NRAS*, *BRAF*, *TP53*, and *DIS3* that are among the most frequent mutated genes in MM. These mutations have an impact on key signaling pathways that control cell survival, proliferation, and drug resistance, including MAPK/ERK, PI3K/AKT, and NF-ҡB (67, 73). In addition, copy number gains/amplifications or deletions of distinct chromosomal regions are acquired in the course of myeloma progression and further promote the clonal evolution of malignant plasma cells. In this regard, alterations in RAS or TP53 genes, as well as the 1q21 chromosomal amplification, are important drivers in the clonal selection process and are detected more frequently after MM treatment (67, 73). Patients treated with high-dose melphalan had the highest rate of tumor mutations (73). Clonal evolution analysis of matched samples from time of diagnosis and MM relapse revealed that clonal selection was detected in relapsed stages of all patients that had undergone high-dose melphalan treatment (74).

Aside from drug-induced genomic alterations, the genomic instability is also influenced by external or environmental factors. In this regard, focal lesions (FLs) are seen as clonally heterogeneous and spatially distributed tumor foci that can be found in up to 84% of MM patients at time of diagnosis. The presence of FLs is linked to myeloma dissemination, disease relapse and adverse outcomes (75). For this reason, FLs are considered as mutational “hot spots” in MM, consistent with the regional outgrowth of advanced tumor clones (13, 75, 76). The increased acquisition of mutations within FLs might generate subclones with enhanced capacities for immune evasion, drug resistance and dissemination thus driving disease progression. Hypoxic tension within the tumor microenvironment and (epi-) genetic changes are associated with MM resistance and both factors are highly induced within FLs (77–79). In line with these findings, multi-region sequencing of matched and synchronously taken iliac crest and FLs biopsies from MM patients revealed spatial clonal heterogeneity in MM. Somatic aberrations such as the deletion del(17p) or *MYC* translocations – alterations that are frequently found in advanced MM stages - were exclusively present at one biopsy site only. In contrast, early genetic alterations such as t(4;14) were uniformly detected at both sites suggesting spatio-temporal evolution from a common subclone (13, 14, 80). Moreover, the longitudinal analyses of FLs and matched samples at later onset of MM relapse showed a high similarity in the clonal composition between both manifestations further supporting the role of FLs as mutational “hotspots” and origins of clonal evolution (14).

**Figure 1:**
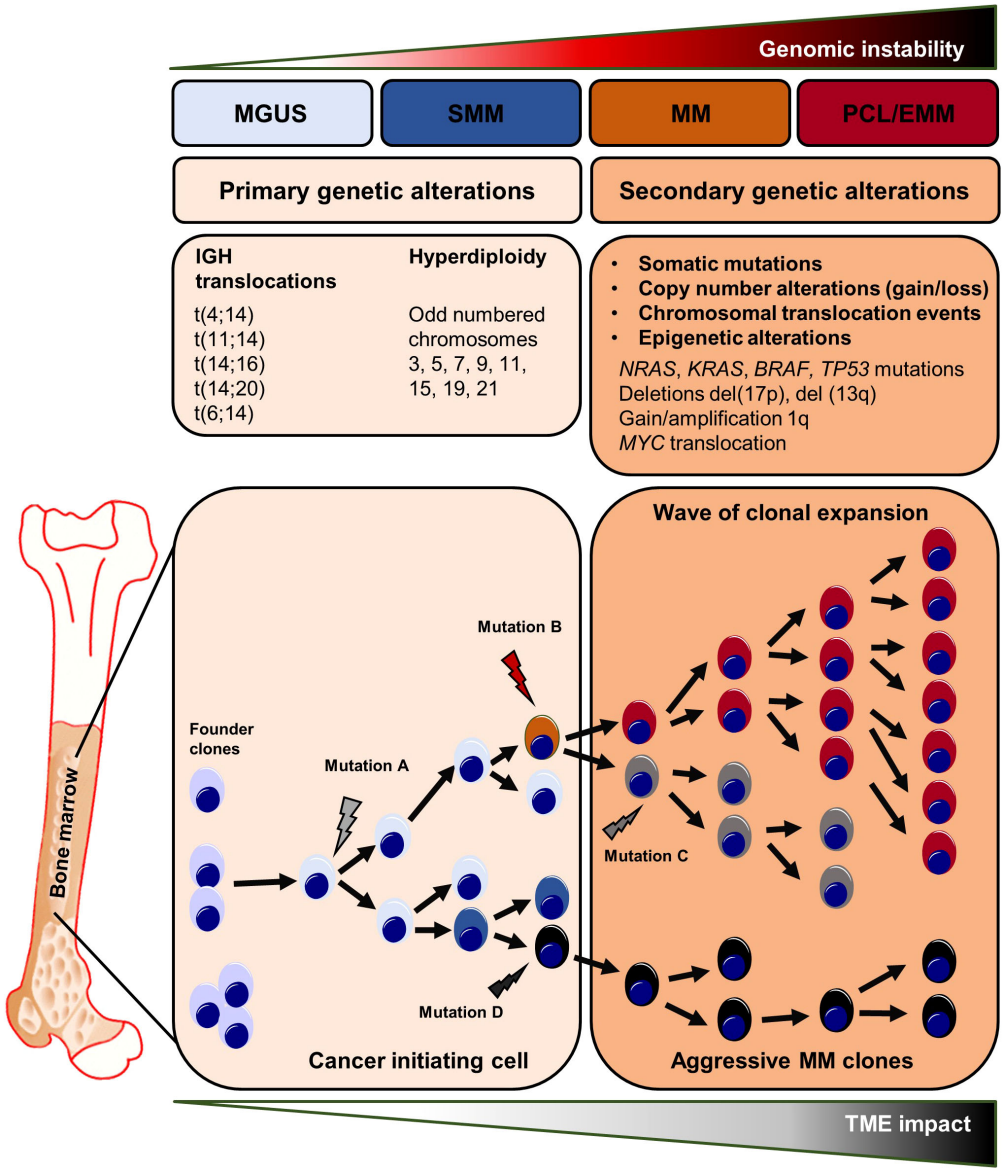
Genetic abnormalities in myeloma initiation and clonal evolution. Primary and secondary genetic processes involved in the development and progression of MGUS and SMM to MM and ultimately to plasma cell leukemia and extramedullary myeloma. Chromosomal translocations are seen at the early steps of the malignant transformation process. The development of MGUS to MM is frequently related with c-MYC overexpression, RAS mutations, and chromosome 13 deletion. The final step, PCL/EMM manifestation, is accompanied by NF-ҡB pathway activating mutations, chromosomal translocation involving the c-MYC gene, 1q gain, loss of 1p and deletion of 17p where the TP53 gene is situated, BRAF mutations, and epigenetic alterations. EMM, extramedullary myeloma; MGUS, monoclonal gammopathy of undetermined significance; MM, multiple myeloma; PCL, plasma cell leukemia; SMM, smoldering multiple myeloma.

Aside from genetic alterations, the clonal evolution process is regulated by epigenetic changes such as DNA methylation and histone modifications. DNA hypermethylation can result in the downregulation/loss of important tumor suppressor genes such as *CDH1* or *SHP1* or alter the expression of genes that influence the sensitivity towards anti-myeloma drugs (81–84). The translocation t(4;14) leads to Nuclear Receptor Binding SET Domain Protein 2 (*NSD2*) overexpression with subsequent NSD2-driven epigenetic downregulation of specific genes that desensitize affected cells for bortezomib therapy (85, 86). Epigenetic alterations can not only affect myeloma cells but also occur in the cellular components of the TME, especially in bone marrow stromal cells (BMSCs) (87, 88). Direct co-culture of BMSCs from healthy individuals together with the myeloma cell line MM.1S has been reported to induce DNA methylation changes in BMSCs. These epigenetic modifications in BMSCs were predominantly located in osteogenic gene loci, regulating the differentiation process of BMSCs to osteoblasts. Thus, myeloma cells might inhibit the formation of new bone-forming osteoblasts that have been proposed to keep myeloma cells in quiescent states or induce their apoptosis (16, 89, 90). In this regard, separated transwell-based cultures of BMSCs and myeloma cells resulted in similar epigenetic alterations in BMSCs as direct co-culture experiments suggesting that soluble factors induce epigenetic changes in surrounding stromal cells thus creating a tumor-supportive microenvironment stimulating myeloma cell growth and survival (90). As a consequence, targeting of enzymes that are involved in DNA methylation (i.e. DNA methyltransferases or histone methyltransferase G9a) led to significantly lower tumor burden and reduction of bone lysis in immunodeficient mice injected with MM cell lines (90). The genomic instability and multistep molecular pathogenesis involved in MM clonal evolution and expansion are illustrated in **Figure 1**.

## Autocrine signaling pathways involved in the clonal expansion of malignant plasma cells

The growth and survival of malignant plasma cells is highly dependent on the abundance of defined cytokines and growth-promoting factors that are primarily provided by the BM microenvironment. However, myeloma cells can also produce some of these soluble factors by themselves thus stimulating their proliferation or preventing spontaneous and stress-induced apoptosis. In this regard, autocrine signaling may emerge from the release of cytokines and the simultaneous expression of the cytokine-specific receptor on the same cell. Furthermore, the co-expression of growth-stimulatory ligand-receptor pairs may promote the activation of autonomous signaling (91). Clonal subsets that co-express a growth-promoting ligand with its corresponding receptor might harbor a crucial selection advantage. Exemplary, amplification of the chromosomal region 1q21 - frequently detected in advanced disease stages - is often accompanied by the expression of the IL-6 receptor (whose encoding gene is located on the 1q21 region) (92). IL-6 receptor (IL-6R) expression is reportedly associated with a poor prognosis in MM (93). Moreover, myeloma cell lines that express the IL-6R respond positively to induction of IL-6R signaling, with increased proliferation and reduced apoptosis (94, 95). The expression levels of microRNAs such as miR-197-3p and miR-451 influence bortezomib resistance by inhibiting the IL-6/IL-6R signaling pathway (94, 96). In addition, the availability of IL-6 is crucial for the survival and treatment sensitivity of malignant plasma cells (97). Multiple studies have shown that malignant plasma cells can generate and secrete IL-6 hence promoting their own survival (98–100). IL-6 enhances pro-survival signaling and decreases bortezomib sensitivity because it activates STAT3, which acts as a transcription factor for the anti-apoptotic proteins Bcl-2 and Bcl-xL (97, 101). Taken together, IL-6R expressing subclones might undergo a positive selection process based on their enhanced responsiveness to IL-6 that is provided by the TME or secreted by themselves subsequently preventing spontaneous or stress-induced cell apoptosis. In line, Ryu et al. reported frequent IL6/IL-6R co-expression in extramedullary MM that no longer require IL-6 provided by the BM niche (102). Approaches to target the IL-6/IL-6R signaling pathway have been tested in a cohort of transplant-eligible newly diagnosed MM patients using the anti-IL6 antibody siltuximab in combination with lenalidomide, bortezomib and dexamethasone (**Table 1**). Complete remission or very good partial remission were achieved in 2/11 (18%) and 2/11 (18%) of patients, respectively (103).

**Table 1:** Overview clinical studies that target MM autocrine and TME-mediated signaling pathways.

	Signaling Pathway	Drug	Mechanism of Action	Clinical Phase	Disease	Status	Outcome: CR/VGPR/PR Case/cohort (%)	NCT Number	Ref
Autocrine Signaling	IL-6/IL-6R	Siltuximab(CNTO 328)	Anti-IL-6	Phase I/II	NDMM	Completed	**2/11 (18%)** **2/11 (18%)** **6/11(55%)**	NCT01531998	(103)
		Toclizizumab	Anti-IL-6R	Phase I	RRM	Recruiting	-	NCT05391750	-
	IGF-1/ IGF-1R	*AVE1642*	Anti-IGF-1R	Phase I	RRM	Completed	**1/11 (9%)** **0/11 (0%)** **1/11(9%)**	NCT01233895	(104)
		*ASP7487(OSI-906)*		Phase I/II	RRM	Terminated	**1/18 (sCR) (6%)** **3/18 (17%)** **7/18 (39%)**	NCT01672736	-
	FGF/FGFR	AZD4547	FGFR TKI	Phase II	RRM & other cancer types with FGFR aberrations	Completed	**0/48 (0%)** **0/48 (0%)** **4/48 (8%)**	NCT04439240	(105)
		Masitinib (AB1010)	FGFR TKI	Phase III	RRM with t(4;14)	Terminated	–	NCT01470131	–
	Wnt/β-catenin	DKN-01	Anti-DKK1	Phase I	MM/Advanced solid tumors	Completed	–	NCT01457417	(106, 107)
	BAFF	Tabalumab (LY2127399)	Anti-BAFF	Phase II	RRM	Completed	**Cohort: 300mg** **4/74 (5%)** **16/74 (22%)** **23/74 (31%)**	NCT01602224	(108)
	APRIL	BION-1301	Anti-APRIL	Phase I/II	RRM	Terminated	**No objective response**	NCT03340883	(109)
				Phase I/II	IgA Nephropathy	Active	-	NCT03945318	(110)
TME	VEGF	Bevacizumab	Anti-VEGF	Phase II	RRM	Completed	**1/49 (2%)** **8/49 (16%)** **16/49 (33%)**	NCT00473590	(111)
	VLA-4/ VCAM-1	BG00002 (Natalizumab)	Anti-VLA-4	Phase I/II	RRM	Terminated	**-**	NCT00675428	–
	ICAM-1	BI-505	Anti-ICAM-1	Phase I	RRM	Completed	**No objective response**	NCT01025206	(112)

Autocrine and paracrine signaling loops in multiple myeloma plus therapy concepts. APRIL, a proliferation-inducing ligand; BAFF, B-cell activating factor; CR, complete response; DKK1, dickkopf-1; FGF, fibroblast growth factor; FGFR, fibroblast growth factor receptor; ICAM-1, intercellular adhesion molecule 1; IGF-1, insulin-like growth factor 1; IGF-1R, insulin-like growth factor 1 receptor; IL-6, Interleukin-6; IL-6R, Interleukin-6 receptor; MM, multiple myeloma; NDMM; newly diagnosed multiple myeloma; PR, partial response; Ref, reference; RRM, relapsed & refractory myeloma; sCR, stringent complete response; TKI, tyrosine-kinase inhibitor; TME; tumor microenvironment; VCAM-1, vascular cell adhesion protein 1; VEGF, vascular endothelial growth factor; VGPR, very good partial response; VLA-4, very-late antigen-4.

**Figure 2:**
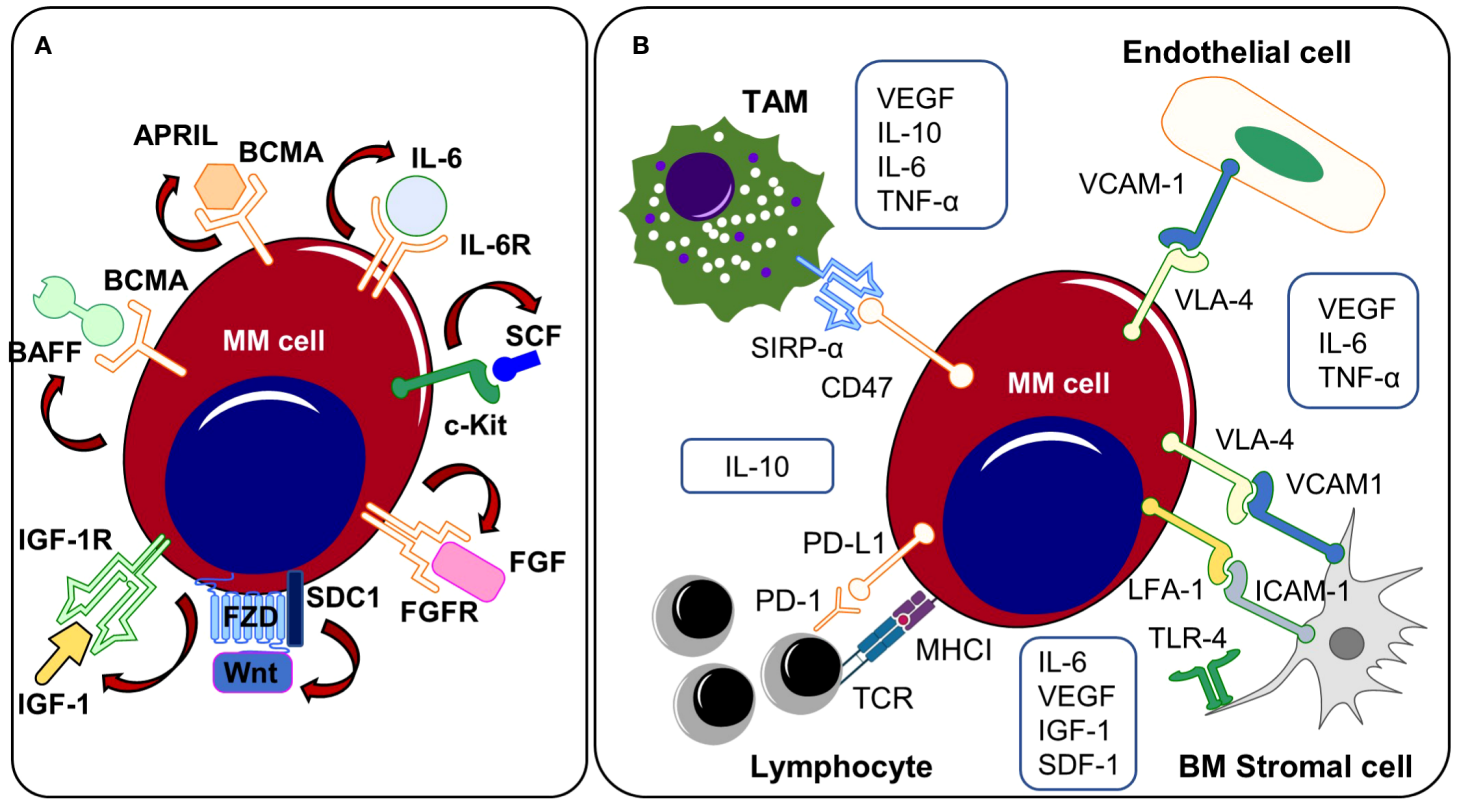
**(A)** Autocrine signaling and **(B)** TME related mechanisms. Both autocrine signaling cascades and interactions with the TME promote the outgrowth of clonal subsets that (co-)express growth promoting ligands/receptors on the cell surface. APRIL, a proliferation-inducing ligand; BAFF, B-cell activating factor; BCMA, B cell maturation antigen; BM, bone marrow; c-Kit, receptor tyrosine kinase; FGF, fibroblast growth factor, FGFR, fibroblast growth factor receptor; ICAM-1, intercellular adhesion molecule 1; IGF-1, insulin-like growth factor 1; IGF1R, insulin-like growth factor 1 receptor, IL-6, Interleukin-6; IL-6R, Interleukin-6 receptor; IL-10, Interleukin-10; LFA-1, lymphocyte function-associated antigen 1; MM, multiple myeloma; PD-1, programmed Cell Death 1; PD-L1, programmed Cell Death Ligand 1; SCF, stem cell factor; SDC1, Syndecan 1, SIRPα, signal regulatory protein α; SDF-1, stromal cell-derived factor 1; TAM, tumor-associated macrophage; TLR-4, toll-like receptor 4; TME, tumor microenvironment; TNF-α, tumor necrosis factor alpha. VCAM-1, vascular cell adhesion protein 1; VEGF, vascular endothelial growth factor; VLA-4, very-late antigen-4.

Several other autonomous and autocrine signaling pathways play a key role in the expansion of clonal plasma cells (**Figure 2A**). Expression of the insulin-like growth factor 1 receptor (*IGF1R*) was described in the majority of patients with extramedullary disease manifestation and positive expression profiles were associated with the occurrence of the high-risk cytogenetic alterations t(4;14), t(14;16) and were linked to reduced overall survival suggesting a potential selection advantage of IGF1R positive clones in the course of MM progression (113). Indeed, *IGF1R* and *IGF1* are frequently co-expressed in myeloma cells and Chiron et al. discovered that blocking IGF1R signaling inhibits the self-renewal of myeloma cells *in vitro*. In addition, inhibition of self-renewal and colony formation in certain myeloma cell lines after treatment with a c-KIT receptor antagonist indicates the presence of a SCF/c-KIT autocrine signaling loop (114). Myeloma expansion is further regulated by the Wnt/β-catenin signaling pathway that is often over-activated in MM, partially driven by the autocrine release of Wnt ligands and/or by the (over-) expression of distinct molecules such as Syndecan-1 (CD138) or Leucine Rich Repeat Containing G Protein-Coupled Receptor 4 (LGR4) on cancer cells (44–46, 115). Wnt-ligands can directly bind to heparan sulfate side chains of Syndecan-1 thus mediating abnormal Wnt-signaling activity in MM. Indeed, CRISPR/Cas9-mediated deletion of Syndecan-1 heparan sulfate side chains inhibits Wnt-signaling activity and reduces the growth of MM cell lines suggesting autonomous Wnt-signaling stimulation via the autocrine release of Wnts (45). Malignant plasma cell clones may prevent both spontaneous and stress/drug-induced apoptosis by autocrine secretion of various pro-survival factors such as TNF-α, MIP-1, sonic hedgehog or GAS6 (116–119). Autocrine TNF-α/MCP-1/TNF-R2 signaling further enhances trans-endothelial migration of MM cell lines and primary myeloma samples (120, 121). B-cell activating factor (BAFF) and a proliferation inducing ligand (APRIL) are essential survival factors for myeloma cells (122). While these cytokines are primarily produced by cells of the BM microenvironment, previous research has revealed that malignant plasma cells may express and release BAFF and APRIL in an autocrine manner (116, 122). The translocation event t(4;14) (p16.3;q32) results in the overexpression of the fibroblast-growth factor receptor 3 (FGFR3) and can be detected in about 15% of all myeloma patients (61, 123). Different studies demonstrated that myeloma cells produce FGF and that autocrine FGF/FGFR signaling prevents oxidative stress-induced apoptosis (124, 125). In line, t(4;14) is seen as a high-risk cytogenetic alteration that occurs in advanced MM stages and FGFR expression might promote the positive selection of t(4;14) carrying subclones. Therapeutically, tyrosine kinase inhibitor-mediated FGF/FGFR signaling disruption abrogated growth and dissemination of MM cell lines *in vivo* (125). In addition, treatment approaches targeting FGF/FGFR signaling have been tested in phase 3 clinical trials using the tyrosine kinase inhibitor masitinib. Other monoclonal antibodies or small molecules targeting autocrine signaling pathways or MM-promoting components of the TME are currently tested in phase 1 and 2 clinical trials.

In summary, genomic instability with the acquisition of specific secondary mutations might lead to the expression of distinct ligand-receptor pairs in myeloma subclones inducing cell proliferation or protecting from spontaneous and/or drug-induced apoptosis thus favoring the expansion of the latter.

## Impact of the tumor microenvironment on clonal expansion and dissemination

Malignant plasma cells are surrounded by a variety of cell types and extracellular matrix in the BM. These elements work together to form a cytokine-rich and growth-promoting micromilieu or “niche” known as the tumor microenvironment. The TME is composed of the cellular fractions (e.g., fibroblasts, endothelial cells, osteoblasts, osteoclasts, hematopoietic cells, immune cells and neuronal cells). In addition, the extracellular matrix and its liquid milieu (cytokines, chemokines, and growth factors) are also essential components (16, 126–128). The TME promotes the survival and expansion of myeloma cells via different mechanisms. Furthermore, it has a crucial impact on the genomic instability in MM (129). In general, the BM environment is characterized by low oxygen levels (130). Due to the rapid expansion of malignant plasma cells within the BM during MM progression and the generation of abnormal neo-vessels, the oxidative stress within the TME increases. The latter may lead to the accumulation of reactive oxygen species (ROS), which can cause DNA damage. During the anti-tumor response, activated immune cells of the TME additionally release ROS, further exacerbating genomic instability in cancer cells and generating possible selection advantages (131, 132). In addition, hypoxia has been shown to induce myeloma cell dissemination and impair treatment sensitivity (77, 78, 133, 134). Hypoxia-inducible factors (HIFs) can alter the transcription of key regulators involved in cancer stemness, cell proliferation, drug resistance and/or cell survival (37, 77, 135). Moreover, increased *HIF1A* gene expression was detected in end-stage extramedullary MM cases (102). Taken together, areas with low oxygen levels might be “hotspots” for the generation of new clonal subpopulations that are prone to disseminate to distant BM sites and lead to MM progression.

While subcutaneously injected MM cell lines do not enter the blood circulation or disseminate in immunodeficient mice, myeloma cells that are grown in subcutaneously implanted bone chips maintain their capacities to disseminate and re-engraft at distant BM sites, indicating that the interactions with specific compartments of the BM TME play a fundamental role in MM spread and progression, as previously shown by Shen et al. (12). Here, they found that the clonal selection of myeloma cells occurs primarily in distant BM sites by using an implanted bone chip xenograft model combined with a fluorescence-based tracking system of clonal subsets enabling the simultaneous assessment of myeloma cell dissemination and clonal heterogeneity. While the primary tumor site showed a high degree of clonal heterogeneity with the co-occurrence of multiple clonal subsets, distant BM metastases displayed a markedly reduced heterogeneity with predominance of a single clonal population. Compared to myeloma cells from the primary site, disseminated clusters were characterized by an enrichment of genes that are linked to MM progression, suggesting that only a small number of plasma cell clones harbor the capacities to leave the primary tumor and disseminate to distant BM sites (12). In this regard, various direct and indirect interactions with the TME might promote the outgrowth and dissemination of single plasma cell clones (**Figure 2B**). The growth promoting cytokines IL-6 and APRIL are provided by different sources of the TME including bone marrow stromal cells (BMSCs), perivascular cells, eosinophils, mast cells and megakaryocytes (128, 136–140). Apart from IL-6, BMSCs additionally produce VEGF, IGF1 or SDF-1, soluble factors that have been shown to directly or indirectly affect myeloma growth, migration and invasion (141–144). Induction of the Toll-like receptor-4 (TLR-4) that has been found overexpressed on MM-BMSCs leads to the release of IL-6 by BMSCs, which promotes cell growth and survival in MM cells. Targeting these pathways has been demonstrated to offer therapeutic potential for the management of multiple myeloma. In this regard, selective blockade of TLR-4 reduced myeloma progression in murine myeloma models (145). Interleukin-10 (IL-10) is an anti-inflammatory cytokine that is produced by macrophages and lymphocytes (146, 147). In newly diagnosed MM patients, elevated IL-10 serum levels negatively correlated with both, therapy response and overall survival suggesting an apoptosis preventing function of IL-10 (147). M2 polarized tumor-associated macrophages (TAMs) promote myeloma progression via the release of multiple soluble factors and cytokines that directly influence cell growth (148). Clodronate-based depletion of M2 TAMs in xenograft-bearing nude mice resulted in significantly decreased tumor growth and reduced microvessel density. VEGFA serum levels were significantly lower in M2 depleted mice (148). Moreover, release of IL-6 and TNF-α by TAMs increases the vascular leakiness of newly formed tumor vessels in Vk*MYC myeloma mice thus facilitating the entrance of single plasma cell clones into the blood circulation (149). TNF-α has been shown to not only affect the vessel wall permeability but also directly enhances trans-endothelial myeloma cell migration (121).

Aside from cytokine or growth-factor driven mechanisms, direct cell-cell and cell-matrix interactions mediated through surface molecules and adhesion receptors play a critical role in MM expansion and dissemination. In advanced MM, platelets are highly activated and have been reported to enhance myeloma proliferation and engraftment through an IL-1β dependent mechanism (150). Myeloma cell adhesion to BMSCs or extracellular matrix components by expression of adhesion molecules such as Syndecan-1, intercellular adhesion molecule 1 (ICAM-1) or vascular cell adhesion protein 1 (VCAM-1) prevent apoptosis, resulting in cell-adhesion driven drug resistance (151–156). Myeloma cells express high levels of very late antigen-4 (VLA-4)/integrin α4β1 that binds to its receptor ICAM-1 expressed on endothelial cells. The VLA-4/ICAM-1 interaction enables myeloma cell adhesion to the vessel wall followed by trans-endothelial migration and engraftment (157, 158). Therapy concepts that prevent the vicious circle of myeloma cell dissemination and re-engraftment are currently tested in pre-clinical studies. Injection of VLA-4 deficient 5TGM1 murine myeloma cells into syngeneic recipients leads to higher extra-and reduced intramedullary disease burden with improved survival (158). Targeting VLA-4 with nanoparticles overcomes cell adhesion mediated drug resistance in myeloma cell lines and enhances chemotherapy response in 5TGM1 myeloma bearing mice (159).

## Mechanisms of immune evasion favoring MM clonal selection

Cancer cells are targeted by immune cells and thus have developed strategies to evade the host’s immune response. Both, direct mechanisms - such as upregulation of immune checkpoints on cancer cells – and indirect mechanisms (e.g. an overall immunosuppressive tumor microenvironment) are involved in cancer cell immune evasion. As direct mechanisms, upregulation of the immune checkpoints PD-L1 (CD274) and CD276 have been reported in MM patients (160–162). In addition, PD-1, an activation and exhaustion marker, is present on CD8^+^ T cells invading myeloma foci (163). In advanced MM stages, activating mutations in MYC oncogenes are frequently detected (164). In genetically engineered MM mouse models, early transgenic activation of the MYC oncogene was associated with enhanced numbers of MM infiltrating PD-1^+^ TIGIT^+^ LAG3^+^ CD8^+^ T cells, whereas late MYC activation was linked to significantly decreased numbers of activated/exhausted CD8^+^ T cells (165). Accordingly, anti-PD-1 therapies significantly reduced MM burden in mice with early transgenic MYC activation but had no effect on disease burden in mice with late oncogenic MYC activation. Moreover, pharmacological inhibition of MYC resulted in a downregulation of PD-L1 expression in malignant plasma cell clones upon early transgenic MYC activation, indicating that early myeloma subclones with alterations in the MYC oncogene may express PD-L1 thus preventing their elimination by PD-1^+^ CD8^+^ cytotoxic T cells (165). Further, HIF1α has been found to induce PD-L1 in cancer cells and might thus provide an advantage to myeloma subclones that are exposed to hypoxic tension in the BM or at extramedullary sites (102). In addition, the expression of IL-32γ by myeloma cells has been reported to induce the PD-L1 expression on tumor-associated macrophages, which suppresses CD8^+^ effector T cells. Increased IL-32γ expression levels were predominantly observed in relapsed MM stages, indicating that IL-32γ may be upregulated in clonal populations during the progression of MM and provide a selective advantage based on its immunosuppressive effect on infiltrating immune cells (166). Moreover, myeloma cell clones may directly prevent phagocytosis by TAMs via upregulation of the “don’t eat me” molecule CD47, which binds to the inhibitory receptor SIRPα expressed on macrophages (167). In preclinical studies, CD47 blockade resulted in increased phagocytosis and reduced growth of CD47 expressing myeloma cell lines both *in vitro* and *in vivo* (167). Clinical trials are currently conducted to test the efficacy of the anti-CD47 monoclonal antibody magrolimab in combination with other anti-myeloma drugs in relapsed and therapy refractory MM patients (168). In addition, natural killer (NK) cells were found to be deficient in perforin, CCLA5, and granzyme B and myeloma cells have been shown to directly induce inhibitory molecules in NK cells (e.g. by the expression of leukocyte immunoglobulin-like family proteins) (66, 102).

Aside from mechanisms that are mediated by myeloma cells to directly inhibit effector cell functions, an immunosuppressive tumor microenvironment – in parts induced by cancer cells themselves - may further promote MM immune evasion. Malignant plasma cells can promote the expression of CD84 on cells of the TME by releasing macrophage migration inhibitory factor (MIF). CD84 upregulation induces the differentiation and expansion of myeloid-derived suppressor cells (MDSCs), which inhibit T cell activity (169). IL-10 and TGFβ, primarily released by cells of the TME, have been shown to inhibit T cell mediated immunity (170–172). However, malignant plasma cells also secrete TGFβ by themselves thus creating an immunosuppressive environment facilitating immune evasion and expansion (173). Autocrine and/or paracrine IL-6 secretion induces IL-10 production, further suppressing T cell function, hence promoting myeloma clonal expansion (174–176). IL-10 may also affect TAM polarization towards an immunosuppressive M2 phenotype (176). The production of IL-10 and TGFβ by regulatory T cells (Tregs) has been shown to mediate their immunosuppressive activity on T effector cells in MM (177–179). MDSC numbers in MM patients’ BM are significantly higher and they have been shown to induce Treg differentiation and promote myeloma clonal selection and progression (128, 180–182). Furthermore, Leone et al. reported that dendritic cells accumulate in the BM of myeloma patients, where they promote cancer cell immune evasion by downregulating proteasome subunits (183). Therefore, understanding how immunosurveillance influences clonal selection in MM is crucial. By unraveling the dynamic interactions between the TME, immune system and malignant plasma cells, we can gain insights into the mechanisms underlying the immunosurveillance- related clonal evolution in MM.

## Clonal selection by MM therapy

**Figure 3:**
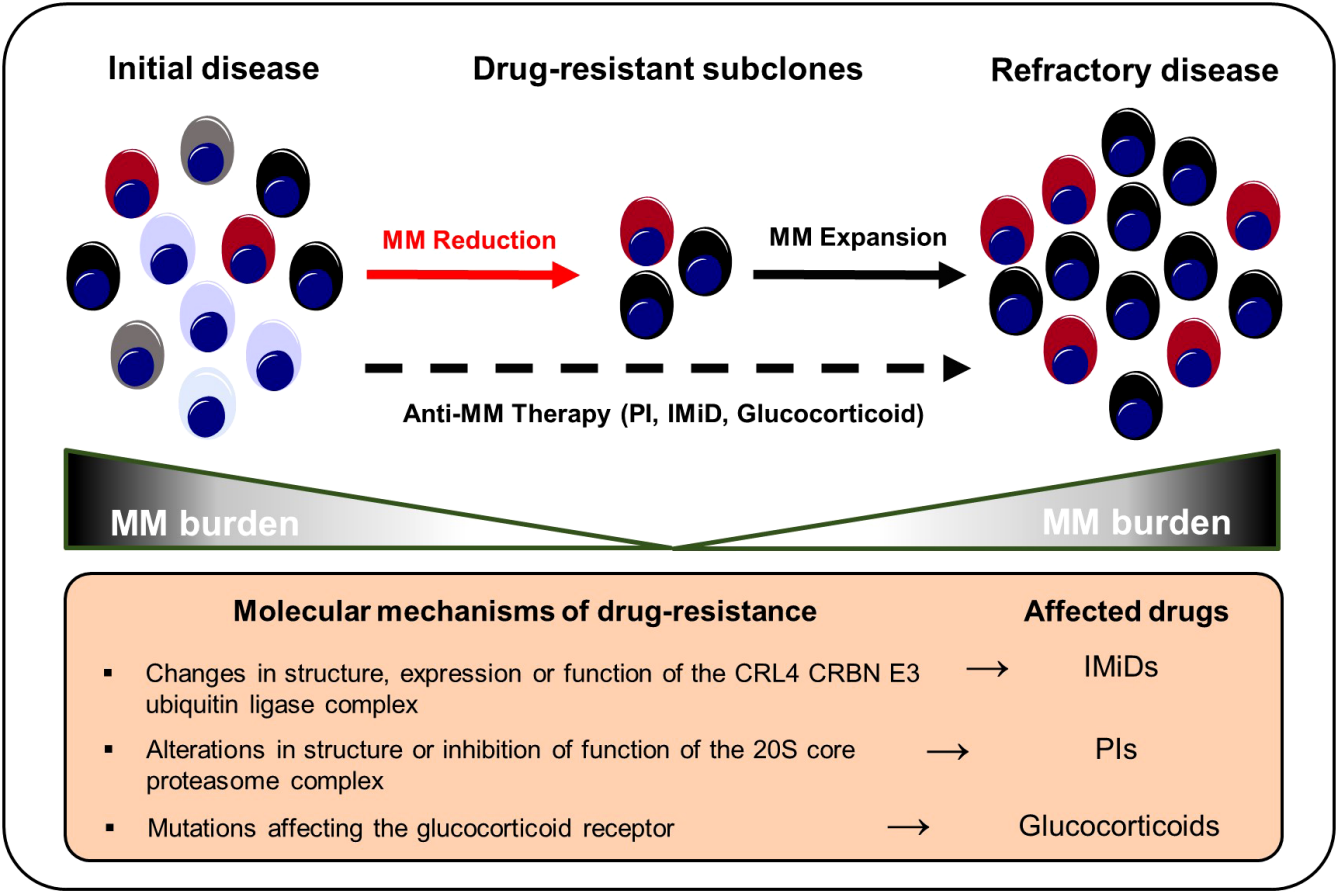
Selection and expansion of drug-resistant subclones under anti-MM therapy. Genetic instability increases the repertoire of drug-resistant subclones in the course of MM progression. Anti-myeloma therapies result in a selection of therapy-refractory and more aggressive clonal subsets that ultimately result in MM recurrence. IMiD, immunomodulatory imide drug; MM, multiple myeloma; PI, proteasome inhibitor.

Therapy resistance is a fundamental challenge in the management of MM. Although the majority of MM patients responds well to first-line therapies, almost all patients eventually experience MM recurrence and therapy-refractory disease states (184). Based on the acquisition of cytogenetic alterations or distinct mutations, a small proportion of malignant plasma cell clones might become resistant to standard MM therapies such as proteasome inhibitors (PIs), immunomodulatory imide drugs (IMiDs), glucocorticoids, and monoclonal antibodies and subsequently outcompete drug-sensitive populations (**Figure 3**). Misund et al. performed whole exome and RNA sequencing analyses for purified CD138^+^ cells from paired MM patients before and after therapy with different anti-myeloma drugs to compare genomic and transcriptomic changes as well as clonal evolution under treatment pressure. Changes in the clonal composition occurred in 82% of patients under therapy and alterations were primarily detected among *RAS*, 1q21 and *TP53* (73). Ryu et al. detected overexpression of proteasome components in clonal subpopulations from relapsed and therapy refractory MM patients that had received bortezomib-containing combination therapies indicating selection and outgrowth of PI-resistant subclones under therapy (102). Corre et al. investigated the clonal heterogeneity among patients that had undergone a homogeneous treatment regimen of VTD (bortezomib, thalidomide and dexamethasone) followed by melphalan. Here, the frequencies of *KRAS*, *NRAS* and *TP53* mutations were increased in relapsed MM patients compared to initial manifestations suggesting an induction of mutations by the treatment itself or increased resistance of mutation harboring subclones against VTD (185). Deletion of the chromosomal region 17p (del17p) is considered a high-risk cytogenetic alteration and is associated with mono-allelic loss of *TP53* and shorter overall survival rates (186, 187). Co-occurrence of del17p with a *TP53* mutation on the second allele – detected in around 6-8% of MM patients at the time of diagnosis - results in a bi-allelic inactivation and complete loss of p53 that is reportedly accompanied with worse outcomes compared to mono-allelic inactivations (187, 188). In line, AMO-1 MM cell line derived clones with bi-allelic *TP53* inactivation out-competed AMO-1 clones with mono-allelic *TP53* aberrations in comparative *in vitro* studies indicating a selection advantage of *TP53* double-hit myeloma cell clones (187, 189). Walker et al. characterized newly diagnosed MM patients with bi-allelic *TP53* aberrations as a distinct high-risk subgroup with rapid disease progression and extremely aggressive behavior despite the use of combination treatments of PIs, IMiDs, glucocorticoids and cyclophosphamide (190). These findings highlight the need to adapt MM risk-stratification criteria and the therapy of choice in accordance to the constellation of both cytogenetic and somatic alterations. However, so far, no specific treatment options are available for high-risk MM patients with *TP53* aberrations (191). For this reason, clinical trials are urgently needed to investigate whether high-risk patients with *TP53* mutations may profit from early intensified therapy regimens involving novel immunotherapies such as bispecific T-cell engagers or chimeric antigen receptor T cells. In addition, further preclinical studies need to be conducted to test the potential of drugs inducing p53-dependent synthetic lethality in MM. In this regard, recent research has shown that myeloma cells with p53 deficiency are more vulnerable to Chk1 inhibition compared to p53 proficient cancer cells (192).

Various tumor-related mechanisms that promote anti-myeloma drug resistance have been described. Thalidomide has been shown to inhibit tumor angiogenesis and dampen inflammation thus counteracting the release of cytokines such as TNF-α that are essential for myeloma cell growth (193). In addition, IMiDs also directly affect myeloma cell survival by binding to Cereblon (CRBN) – a component of the CRL4^CRBN^ E3 ubiquitin ligase complex (CUL4–ROC1–DDB1–CRBN). Subsequent ubiquitination and degradation of the transcription factors Ikaros (*IKZF1*) and Aiolos (*IKZF3*), which control myeloma survival and proliferation genes (e.g., *MYC* or *IRF4*), result in growth limitation and death (194–198). Changes in the structure, expression or function of CRL4^CRBN^ E3 may generate IMiD resistant clonal plasma cell populations. Overall, IMiD resistance affects 10-20% of relapsed myeloma patients (196, 198–200). *CRBN* and *CUL4* mutations prevent IMiDs from binding to CRL4^CRBN^, reducing IMiD efficiency (201). Lenalidomide-refractory MM patients had a higher incidence of *COP9* signalosome gene loss, whose products are essential for CUL4-ROC1-DDB1-CRBN E3 ubiquitin ligase maintenance and activity. Approximately, 16% of IMiD patients lost the *COP9* signalosome gene region on chromosome 2q37 while none of the patients in the control arm developed a 2q37 chromosomal aberration (202). MM patients receiving IMiD-based therapy had a greater prevalence of *IKZF1* mutations (196). Ng et al. showed that overexpression of CDK6 in MM cell lines increases lenalidomide and pomalidomide resistance. Inhibition of CDK6 kinase functions, on the other hand, increases IMiD sensitivity. In addition, relapsed MM bone marrow samples from lenalidomide-pretreated individuals showed CDK6 upregulation. Therapeutically, the combination of pomalidomide with palbociclip (a CDK4/6 inhibitor) increased the overall survival of MM.1S tumor-bearing mice (198).

Glucocorticoids are a fundamental pillar in most MM therapy regimens (203). Glucocorticoids have been shown to trigger apoptosis in myeloma cells by suppressing anti-apoptotic genes such Bcl-xL and nuclear factor kappa B (NF-ҡB) (203, 204). However, malignant plasma cell clones may develop resistance to glucocorticoids like dexamethasone and prednisone. Dexamethasone and prednisone’s pro-apoptotic and anti-proliferative effects are blocked by genetic anomalies and/or mutations occurring in the glucocorticoid receptor *NR3C1* in relapsed multiple myeloma patients. Genetic alterations in *NR3C1* undermine the NF-ҡB mediated pro-apoptotic and anti-proliferative actions of dexamethasone and prednisone thus favoring *NR3C1*-mutation harboring clones (201, 204). Overexpression of MDR1 and Survivin (*BIRC5*), as well as downregulation of the apoptosis activator BIM (*BCL2L11*) were found in established dexamethasone-resistant MM cell lines. MDR1 inhibition or Survivin knockdown re-sensitized myeloma cells for dexamethasone (205).

PIs target the proteasome 20S subunit beta 5 (PSMB5). Point mutations affecting *PSMB5* or other components of the proteasome complex play a central role in MM drug resistance (206). Aside from that, other resistance mechanisms to PIs (e.g., bortezomib and carfilzomib) have been described. PI resistance is frequently associated with chromosome 1q21 gain or amplification occurring in advanced MM stages. Various genes that are located at the 1q21 chromosomal region reportedly influence bortezomib sensitivity such as *PSMD4* (207–209). Overexpression of interferon-stimulated 20 kD exonuclease-like 2 (*ISG20L2*) on chromosome 1q is associated with poor response owing to its high affinity in binding bortezomib (BTZ) and inhibiting the proteasome complex (210). In addition, carfilzomib sensitivity in MM cell lines is reduced by increased 1q21 S100 family overexpression localized within the 1q21 region (211). Overexpression of SRC-3 causes BTZ resistance via interacting with NSD2. SI-2 disrupted NSD2-induced SRC-3 stability to overcome BTZ resistance in t(4;14)-positive MM cell lines (212). Hypoxia has been reported to induce SENP1, which increases SRC-3 stability and thereby enhances PI resistance (134).

Immunotherapies and targeted treatments are being developed to overcome therapy resistance and improve the prognosis for patients with relapsed or refractory MM. CD38 has multiple functions in MM cells and immune cells such as promoting tumor cell proliferation, cell adhesion, and survival (213). In MM patients, monoclonal antibodies (mAbs) targeting CD38 (e.g., daratumumab), bi-specific mAbs or chimeric antigen receptor T (CAR-T) cells against the B cell maturation antigen (BCMA) or G protein–coupled receptor, class C, group 5, member D (GPRC5D) have been proven beneficial, particularly in advanced disease stages (**Table 2**) (3, 4, 224). While normal non-lymphoid tissues lack SLAMF7 expression, normal and malignant plasma cells uniformly express high levels of SLAMF7 making it a promising target for immunotherapies (225). SLAMF7 belongs to the group of the signaling lymphocytic activation molecule (SLAM) transmembrane receptors and has been functionally linked to promoting myeloma cell expansion (226, 227). Elotuzumab – an antibody-dependent cellular cytotoxicity (ADCC) anti-SLAMF7 mAb – has been approved for MM treatment (228). In addition, the efficacy of SLAMF7-CAR-T cells has been tested in preclinical studies resulting in the initiation of currently running Phase I/II clinical trials (226, 229).

**Table 2:** Immunotherapies in MM.

	Target	Drug Name	Mechanism of Action	Clinical Phase	Treatment Setting (CT/MT)	Co-Therapeutics	CR or better (≥CR)	NCT (Trial)	Ref
Immunotherapies in MM	CD38	Daratumumab	ADCC Ab	Phase III	CT	Dara + Len + Dex	57%	NCT02076009 (POLLUX)	(214, 215)
				Phase III	CT	Dara + Thal + Bor + Dex	39%	NCT02541383 (CASSIOPEIA)	(216, 217)
				Phase II	CT	Dara + Len + Bor + Dex	82%	NCT02874742 (GRIFFIN)	(218)
	BCMA	Teclistamab	BiTe (BCMA/CD3)	Phase I/II	MT	-	39%	NCT04557098 (MajesTEC-1)	(219)
		Idecabtagene vicleucel (ide-cel))	CAR-T cells	Phase III	MT	-	39%	NCT03651128 (KarMMa-3)	(220)
		Ciltacabtagene Autoleucel (cilta-cel)	CAR-T cells	Phase Ib/II	MT	-	83%	NCT03548207 (CARTITUDE-1)	(221)
	GPRC5D	Talquetamab	BiTe (GPRC5D/CD3)	Phase I	MT	-	23% (405μg/kg)	NCT03399799 (MonumenTAL-1)	(222)
				Phase III	CT	Tal + Dara ± Pom	Recruiting/ Ongoing	(MonumenTAL-3) NCT05016778	-
		OriCAR-017	CAR-T cells	Phase I	MT	-	60%	(POLARIS)	(223)

Immunotherapies in MM targeting plasma cell surface molecules. ADCC, antibody-dependent cell-mediated cytotoxicity; BCMA, B-cell maturation antigen; BiTe, bi-specific T cell engager; Bor, bortezomib; CR, complete response; CT, combination therapy, CAR-T, chimeric antigen receptor T; Dara, daratumumab; Dex, dexamethasone; GPRC5D; G protein–coupled receptor, class C group 5 member D; Len, lenalidomide; MM, multiple myeloma; MT, monotherapy; Pom, pomalidomide; Ref, reference; Tal, talquetamab; Thal, thalidomide.

However, most MM patients eventually progress after immunotherapies. In this regard, protein structural changes, immune checkpoint inhibitor overexpression, and/or an immunosuppressive environment may all exert an influence on the patients’ response to immunotherapy treatments (5). Exemplary, BCMA downregulation was identified in approximately 70% of MM patients undergoing BCMA CAR T cell therapy treatment suggesting that specific mutations can result in the loss of BCMA expression on malignant plasma cell clones that are then selected under therapy (5, 230, 231). Indeed, homozygous deletion of *TNFRSF17* - encoding BCMA - results in a complete loss of BCMA expression and has been described in MM patients after BCMA CAR T cell therapy (232). GPRC5D downregulation or loss was found in six patients with progressive disease after initial response to GPRC5D -targeted CAR T cells suggesting a positive selection of GPRC5D non-expressing subclones (4). CD38 expression on malignant plasma cells reportedly decreases upon anti-CD38 daratumumab treatment (233). Moreover, the efficacy of the ADCC - antibody daratumumab is highly dependent on patients’ NK cell functionality. Verkleij et al. recently showed that non-responding MM patients have higher frequencies of TIM-3^+^ HLA-DR^+^ activated/exhausted NK cells and that NK cells are rapidly depleted upon daratumumab treatment initiation (233).

Overall, different tumor-and non-tumor related mechanisms or their respective interactions, promote drug resistance in clonal plasma cells. Those clones with survival advantages such as point mutations that affect drug binding sites or complete downregulation/loss of surface targets eventually outcompete their drug-sensitive counterparts and become the dominant clonal population.

## Conclusion

In conclusion, MM is characterized by an extensive intratumor heterogeneity starting in the earliest phases of the disease. The (epi-) genetic aberrations are the primary driver of the considerable intratumor heterogeneity and clonal evolution seen in MM. However, the TME crucially contributes to the clonal evolution of specific clones that depend on soluble factors such as chemokines or growth factors or cell-cell interactions provided by BM resident cells. The selection process is further supported by IMiDs, proteasome inhibitors and glucocorticoids. Although, the TME in MM is composed of various immune cells, the immune response is often dysfunctional or suppressed. In contrast, immunotherapy with mAbs, bispecific Abs or CAR-T cells select for resistant clones often characterized as antigen-loss variants. The high intratumor heterogeneity that evolves during disease progression and treatment is responsible for the fact that although many very efficacious treatments have been developed recently, MM still remains an incurable disease.

## Author contributions

SF, RR & AFO wrote the manuscript. All authors contributed to the article and approved the submitted version.
